# Temperature- and moisture-dependent studies on alunogen and the crystal structure of meta-alunogen determined from laboratory powder diffraction data

**DOI:** 10.1007/s00269-016-0840-7

**Published:** 2016-09-19

**Authors:** Volker Kahlenberg, Doris E. Braun, Hannes Krüger, Daniela Schmidmair, Maria Orlova

**Affiliations:** 1 Institute of Mineralogy and Petrography, University of Innsbruck, Innrain 52d, 6020 Innsbruck, Austria; 2 Pharmaceutical Technology, Institute of Pharmacy, University of Innsbruck, Innrain 52c, 6020 Innsbruck, Austria

**Keywords:** Alunogen, Meta-alunogen, Dehydration, Gravimetric moisture sorption, Thermal analysis, X-ray powder diffraction, Ab-initio structure determination

## Abstract

Starting from a synthetic sample with composition Al_2_(SO_4_)_3_·16.6H_2_O, the high-temperature- and moisture-dependent behavior of alunogen has been unraveled by TGA measurements, in situ powder X-ray diffraction as well as by gravimetric moisture sorption/desorption studies. Heating experiments using the different techniques show that alunogen undergoes a first dehydration process already starting at temperatures slightly above 40 °C. The crystalline product of the temperature-induced dehydration corresponds to the synthetic equivalent of meta-alunogen and has the following chemical composition: Al_2_(SO_4_)_3_·13.8H_2_O or Al_2_(SO_4_)_3_(H_2_O)_12_·1.8H_2_O. At 90 °C a further reaction can be monitored resulting in the formation of an X-ray amorphous material. The sequence of “amorphous humps” in the patterns persists up to 250 °C, where a re-crystallization process is indicated by a sudden appearance of a larger number of sharp Bragg peaks. Phase analysis confirmed this compound to be anhydrous Al_2_(SO_4_)_3_. Furthermore, meta-alunogen can be also obtained from alunogen at room temperature when stored at relative humidities (RH) lower than 20 %. The transformation is reversible, however, water sorption of meta-alunogen to alunogen and the corresponding desorption reaction show considerable hysteresis. For RH values above 80 %, deliquescence of the material was observed. Structural investigations on meta-alunogen were performed using a sample that has been stored at dry conditions (0 % RH) over phosphorus pentoxide. Powder diffraction data were acquired on an in-house high-resolution diffractometer in transmission mode using a sealed glass capillary as sample holder. Indexing resulted in a triclinic unit cell with the following lattice parameters: *a* = 14.353(6) Å, *b* = 12.490(6) Å, *c* = 6.092(3) Å, *α* = 92.656(1)°, *β* = 96.654(1)°, *γ* = 100.831(1)°, *V* = 1062.8(8) Å^3^ and *Z* = 2. These data correct earlier findings suggesting an orthorhombic cell. Ab-initio structure solution in space group *P* $\overline{1}$, using simulated annealing, provided a chemically meaningful structure model. The asymmetric unit of meta-alunogen contains three symmetry independent SO_4_-tetrahedra and two Al(H_2_O)_6_ octahedra. The polyhedra are isolated, however, linkage between them is provided by Coulomb interactions and hydrogen bonding. In addition to the water molecules which directly belong to the coordination environment of the aluminum cations there are two additional zeolitic water sites (Ow1 and Ow2). If both positions are fully occupied meta-alunogen corresponds to a 14-hydrate. Structural similarities and differences between the previously unknown structure of meta-alunogen and alunogen are discussed in detail. Since hydrous aluminum sulfates have been postulated to occur in Martian soils, our results may help identifying meta-alunogen by X-ray diffraction not only on the surface of the Earth but also using the Curiosity Rover’s ChemMin instrument.

**Electronic supplementary material:**

The online version of this article (doi:10.1007/s00269-016-0840-7) contains supplementary material, which is available to authorized users.

## Introduction

Alunogen is a globally occurring natural hydrous aluminum sulfate. Although it cannot be considered an important rock forming mineral, it is not uncommon and has been found in about 260 localities (http://www.mindat.org, accessed June 3, 2016) including quite a number of different geological settings. The most important ones are associated with the oxidation of metal-sulfide mineral deposits or tailings impoundments in aride climates (Jambor et al. 2000 and references cited therein). However, alunogen has been also observed in efflorescences due to evaporation of sulfate-rich pore waters in shales (Northern Ohio, Carlson and Whitford 2002). It has been described as an alteration product of kaolinite by acid sulfate fluids (Te Kopia geothermal field, New Zealand, Martin et al. 1999) or in incrustations from exhalation-condensation processes related to hydrothermal and gas activities at fumaroles (Soufrière Hills Volcano, Caribbean island of Montserrat, Boudon et al. 1996 or Mutnovsky Volcano, Kamchatka Peninsula, Russia, Bortnikova et al. 2008). Another environment where alunogen comes into play is related to coal mining. The mineral has been discovered in larger quantities in burning coal mining waste dumps or as deposits derived from gases exhaled from surface vents associated with underground coal fires (Stracher 2011). Furthermore, alunogen has been discussed to be a potential constituent of Martian soils (Golden et al. 2005; Wang and Zhou 2014). Notably, the synthetic analogue of alunogen is also of importance for industrial inorganic chemistry. For decades, it has been employed as an efficient flocculant in sewage treatment and purification of water. Further applications include its usage as a reagent for paper manufacturing or for leather tanning (Römpp-Online, https://roempp.thieme.de/roempp4.0/do/Welcome.do, accessed March 24th, 2016).

Alunogen is usually described as a heptadecahydrate, i.e., its composition can be written as Al_2_(SO_4_)_3_·17H_2_O or Al_2_(SO_4_)_3_·(H_2_O)_12_·5H_2_O. Interestingly, the exact amount of water in the formula of alunogen has been a matter of debate for quite some time. Data reported in the literature vary between 16 and 18 water molecules p.f.u. (Meyer 1921; Lausen 1928; Larsen and Steiger 1928; Palache et al. 1951; Bayliss 1964; Barret and Thiard 1965a, b; Náray-Szabó 1969; Menchetti and Sabelli 1974; Fang and Robinson 1976; Chou and Soong 1984; Çilgi and Cetişli 2009; Bai et al. 2011; Wang and Zhou 2014). The structural investigations of Menchetti and Sabelli (1974) as well as Fang and Robinson (1976) proved unequivocally that the crystal structure of alunogen contains several loosely bound water molecules. This “zeolitic” water can be easily released from the structure and is responsible for the slightly variable composition of alunogen when the compound is in contact with air of ambient humidity. However, as discussed in detail by Fang and Robinson (1976), the maximum H_2_O content in the alunogen formula corresponds to 17 molecules p.f.u., representing the limit where all five zeolitic water sites in the asymmetric unit are fully occupied. The existence of alunogen as an 18-hydrate is highly questionable. According to Fang and Robinson, it gained currency in the mineralogical literature due to an incorrect normalization of the chemical analysis presented by Lausen (1928). Surprisingly, however, even nowadays many chemical suppliers declare their commercially available synthetic equivalent to alunogen as an “octadecahydrate”.

In addition to uncertainties concerning the precise amount of water present in alunogen, especially when comparing starting materials from various sources and storage histories, there are also discrepancies related to the temperature stability between the results of thermoanalytical studies originating from different groups. The release of structural water has been reported to be connected with two (Bayliss 1964; Földvári 2011), four (Krauss and Fricke 1927; Náray-Szabó 1969; Bretsznajder and Rojkowski 1969; Chou and Soong 1984; Moselhy et al. 1993; Çilgi and Cetişli 2009) or five (Barret and Thiard 1965a, b) step processes, respectively. Even for those publications claiming the same number of dehydration events, there are sometimes significant differences between the compositions of the proposed intermediate hydrates obtained from thermal decomposition. The suggested compounds with general formula Al_2_(SO_4_)_3_·*n*H_2_O include phases with *n* = 1, 3, 5, 6, 9, 11, 12 and 14, respectively. Furthermore, based on spectroscopic investigations, Buzatu et al. (2016) only recently proposed a direct transformation of alunogen into an amorphous phase upon heating. However, there is at least a consensus that the crystalline end-product of the dehydration is anhydrous Al_2_(SO_4_)_3_, the natural equivalent of which corresponds to the mineral millosevichite. The above-mentioned contradictions may be at least partially explained by observations that the dehydration characteristics of the hydrous aluminum sulfates strongly depend on sample-inherent properties such as degree of crystallinity (Gancy et al. 1981), as well as experimental settings of the TGA/DTA experiments including heating rates (Moselhy et al. 1993), amount of material (Moselhy et al. 1993), water vapor pressure (Barret and Thiard 1965a, b; Gancy 1981; Kömives et al. 1984) or gas atmosphere (air, nitrogen, argon and vacuum) inside the furnace (Gancy et al. 1981).

Knowledge about the stability of alunogen as a function of relative humidity is very limited. Larsen and Steiger (1928) already reported about the sensitivity of alunogen with respect to humidity. Fang and Robinson (1976) mentioned a deterioration of alunogen at ambient temperature conditions when exposed to the atmosphere during periods of low indoor humidity in winter. Within several hours dehydration started with the formation of cracks until finally the originally transparent colorless crystals had transformed into white translucent grains. On the contrary, alunogen crystals exhibited no alteration for periods of several weeks during humid summer time.

A possible candidate for a dehydration product of alunogen under atmospheric conditions is meta-alunogen. This mineral, frequently occurring pseudomorphous after alunogen, has been first described by Gordon (1942) in veins of an altered andesite near Francisco de Vergara, Antofagasta, Chile. The suggested chemical composition of meta-alunogen is Al_2_(SO_4_)_3_·13.5H_2_O. Whereas Gordon (1942) allocated the compound to the monoclinic crystal system, Náray-Szabó (1969) indexed a powder pattern of synthetic meta-alunogen with an orthorhombic unit cell (*a* = 12.25 Å, *b* = 13.95 Å and *c* = 15.95 Å) and claimed that the phase contains 14 water molecules p.f.u. The quality of indexing can be conveniently expressed using the so-called F_N_ figure of merit introduced by Smith and Snyder (1979). In more detail, *F*_*N*_ is defined as *F*_*N*_ = *N*/(*N*_poss_ 〈Δ2*θ*〉) where *N* is the number of the first observed Bragg peaks in the pattern, *N*_poss_ is the number of independent reflections up to the *N*th. observed peak, and 〈Δ2*θ*〉 is the average absolute difference between the observed and calculated 2*θ*_hkl_. *F*_*N*_ is usually reported in the form *F*_*N*_ = value (〈Δ2*θ*〉, *N*_poss_) (Pecharsky and Zavalij 2005). When calculated for Náray-Szabó’s data, the figure of merit *F*_20_ has a very low value of 2.4 (0.046, 181) indicating a low reliability of the suggested powder pattern indexing. Further evidence for a conversion between alunogen and meta-alunogen was reported by Wang and Zhou (2014). The authors could show that the dehydration of alunogen in vacuumed desiccators at temperatures between −12 and 21 °C, as well as pressures ranging from 0.16 to 0.26 mbar, resulted in the formation of meta-alunogen. Notably, according to the International Mineralogical Association the status of meta-alunogen is “questionable”, highlighting the need for a re-investigation clarifying the hydration states of alunogen and meta-alunogen, as well as its transformation pathway.

In summary, there is a considerable lack of information concerning not only the high-temperature behavior of alunogen but also the moisture-dependent evolution of this compound. The main objectives of this investigation were (1) to shed some light on the impact of these two parameters on the stability of alunogen using a combination of different analytical methods and (2) to perform more detailed structural investigations on the dehydration products with a special focus on meta-alunogen.

## Experimental details

For the TGA, the X-ray powder diffraction and gravimetric moisture sorption experiments, a commercial sample of alunogen was used (VWR International, ACS grade). A first inspection under a petrographic microscope revealed that the material was highly crystalline consisting of intensively intergrown, thin-platy single crystals up to 100 μm in diameter. About three grams of this material were carefully milled for 5 min at 35 % relative humidity (RH) using an agate mortar and a pestle. Subsequently, the sample was split into three equal batches which were stored for one week in different desiccators at 0, 43 and 75 % RH, respectively, fixed by phosphorus pentoxide (dry conditions) as well as K_2_CO_3_- and NaCl-saturated solutions. Determination of the absolute water content was performed for the sample equilibrated at 43 % RH using a Karl Fischer coulometric titrator C20 instrument (Mettler Toledo, Switzerland). The average value for a total of five measurements was 16.3 ± 0.3 mol water per mol Al_2_(SO_4_)_3_.

### Thermal analysis

TGA measurements were taken on a Setsys Evolution 2400 apparatus (SETARAM Instrumentation, Caluire, France) using a TGA-DTA1600 sample holder and 100 µl Al_2_O_3_ crucibles. The carrier gas (dry air) flow was set to 20 ml/min. The temperature program started with an isothermal hold (at 25° C) of 1 h, to investigate the effect of the dry carrier gas on the sample. Subsequently, a ramp to 1200 °C at a rate of 2 °C/min, followed by cooling to 25 °C (10 °C/min) was programmed. To allow for quantitative evaluation of the TGA signal, a blank experiment subtraction was performed. The samples were weighed at room conditions (approx. 35 % RH) right after removing them from the three different storage containers used to equilibrate at constant moisture conditions (see above). The mass of the empty crucibles was recorded, too. The TGA device was flushed with dry air, prior to inserting each sample. After loading the samples, the measurements were started immediately. At the end of each experiment, the mass of the remaining sample was measured. To determine the number of water molecules initially present in the sample, the mass difference recorded by the TGA experiment has to be compared to the amount of water-free Al_2_(SO_4_)_3_. This amount in turn was calculated from the mass of the remaining end-product (assumed to be Al_2_O_3_) after the disintegration of aluminum sulfate. This method may underestimate the amount of Al_2_O_3_, as impurities or adsorbed water may be included and, therefore, overestimate the initial amount of water.

### X-ray powder diffraction

**Fig. 1:**
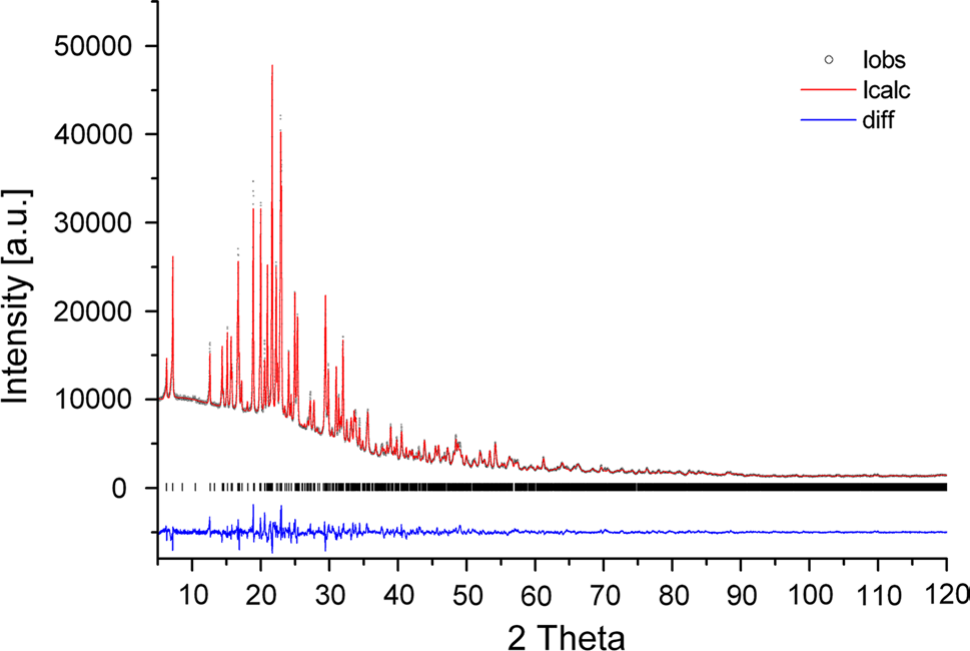
Rietveld plot for meta-alunogen obtained from alunogen by storage at dry conditions (0 % RH). Peak positions permitted by unit-cell metric are indicated by *tick marks* (*middle portion*). Residuals of the Rietveld refinement are *R*_wp_ = 13.59 %, *R*_Bragg_ = 2.45 % and GoF = 2.68

Powder diffraction data for structure analysis have been collected on a Stoe STADI-MP diffractometer operated at 40 kV and 40 mA. The device is equipped with an asymmetric primary beam Ge(111) monochromator (emitting a strictly monochromatic Cu–*K*α_1_ radiation) and a Dectris Mythen1 K microstrip solid-state detector. Diffraction data of the commercial alunogen starting material stored for 1 week at 0 % RH were acquired in Debye–Scherrer geometry. The powder was prepared in a sealed glass capillary of 0.3 mm diameter. In order to prevent partially re-hydration, the whole sample preparation was performed in a glove bag under a constant nitrogen stream. A comparison with the diffraction data presented by Náray-Szabó (1969) suggested that this phase corresponds to the synthetic equivalent of meta-alunogen. For further characterization, individual peaks of the powder pattern were fitted using the WinXPOW software package (Stoe 2004). Indexing of the reflections was done with the CRYSFIRE suite (Shirley 1999) in combination with the program Checkcell (Laugier and Bochu 2004) and resulted in the following unit-cell parameters: *a* = 14.35 Å, *b* = 12.49 Å, *c* = 6.09 Å, *α* = 92.66°, *β* = 96.65, *γ* = 100.83° and *V* = 1062.8 Å^3^ [*F*_20_ = 81.6 (0.007, 36)]. Ab-initio structure determination of meta-alunogen was based on parallel tempering techniques implemented in the program FOX (Favre-Nicolin and Cerny 2002). Assuming the centrosymmetric space group *P* $\overline{1}$ a chemically reasonable model was obtained. For LeBail-fits, subsequent Rietveld analysis as well as for distance and angle calculations the program TOPAS Version 4.2 (Bruker-AXS 2009) was employed. Peak profiles were calculated using Thompson-Cox-Hastings pseudo-Voigt functions including an asymmetry correction. For simulation of the background, Chebyshev polynomials were selected. Soft inter-atomic distance and angle restraints were applied to the SO_4_-tetrahedra and Al(H_2_O)_6_-octahedra, respectively (target values: S–O: 1.46 Å; O–S–O: 109.5°; Al–O: 1.88 Å). A comparison between the resulting observed and calculated step intensities from a Rietveld analysis is given in Fig. 1. A summary of the relevant data collection and refinement parameters can be found in Table 1. Atomic coordinates, isotropic displacement parameters as well as selected interatomic distances and angles for meta-alunogen are listed in Tables 2 and 3. Drawings of structural details have been prepared with the program ATOMS6.4 (Dowty 2011).

**Table 1:** Details of the X-ray powder diffraction data collections and the subsequent Rietveld analysis for meta-alunogen obtained from storage at 0 % RH

Crystal data	
Chemical formula	Al_2_(SO_4_)_3_·13.8H_2_O or Al_2_(SO_4_)_3_(H_2_O)_12_·1.8H_2_O
Unit-cell dimensions	*a* = 14.353 (6) Å
	*b* = 12.490 (5) Å
	*c* = 6.092 (3) Å
	*α* = 92.656 (1)°
	*β* = 96.654 (1)°
	*γ* = 100.831 (1)°
Volume	*V* = 1062.8 (8) Å^3^
Space group type	*P* $\overline{1}$
*Z*	2
Density (calculated)	1.857 g/cm^3^
Intensity measurements	
Diffractometer	Stoe Stadi MP
Instrument geometry	Debye–Scherrer
Monochromator	Primary beam, asymmetric curved Ge(111)
Radiation type, source	X-ray, Cu–*K*α_1_
Data collection temperature	21.5 (5) °C
Generator settings	40 kV, 40 mA
Detector	Dectris Mythen 1 K
Sample container	Glass capillary, Ø 0.3 mm
Range in 2*θ*	5°–120°
Step size	0.01°
Measuring time	20 s/step
Refinement parameters	
No. of structural & profile parameters	146
*R*_wp_, *R*_Bragg_, GoF	13.59 %, 2.45 %, 2.68

**Table 2:** Atomic coordinates and isotropic displacement parameters for meta-alunogen

Atom	*x*	*y*	*z*	*B*_iso_
S1	0.6387 (3)	0.6271 (3)	0.4996 (7)	0.56 (1)
S2	0.8559 (3)	0.3510 (3)	0.4897 (7)	0.56 (1)
S3	0.7340 (3)	0.9893 (3)	0.1955 (7)	0.56 (1)
Al1	0.4013 (5)	0.7195 (6)	0.0399 (11)	0.50 (1)
Al2	0.1041 (5)	0.2764 (5)	0.9678 (11)	0.50 (1)
O1	0.6549 (8)	0.5476 (7)	0.6613 (13)	1.92 (1)
O2	0.7232 (5)	0.7061 (7)	0.4778 (17)	1.92 (1)
O3	0.5665 (5)	0.6877 (7)	0.5691 (19)	1.92 (1)
O4	0.6042 (8)	0.5632 (9)	0.2846 (12)	1.92 (1)
O5	0.9284 (5)	0.2942 (7)	0.4096 (18)	1.92 (1)
O6	0.8469 (9)	0.4447 (6)	0.3567 (16)	1.92 (1)
O7	0.8917 (9)	0.3950 (9)	0.7168 (11)	1.92 (1)
O8	0.7678 (6)	0.2685 (8)	0.4681 (17)	1.92 (1)
O9	0.8084 (6)	0.9629 (9)	0.3645 (14)	1.92 (1)
O10	0.7746 (8)	1.0555 (8)	0.0228 (12)	1.92 (1)
O11	0.6780 (8)	0.8831 (6)	0.0945 (18)	1.92 (1)
O12	0.6643 (5)	1.0396 (8)	0.3124 (15)	1.92 (1)
O13	0.4149 (6)	0.6486 (6)	0.3048 (14)	1.92 (1)
O14	0.3348 (4)	0.5842 (7)	−0.0998 (16)	1.92 (1)
O15	0.2800 (4)	0.7556 (5)	0.0748 (10)	1.92 (1)
O16	0.4655 (4)	0.8557 (6)	0.1606 (13)	1.92 (1)
O17	0.5154 (6)	0.6771 (5)	−0.0331 (10)	1.92 (1)
O18	0.3763 (6)	0.7736 (5)	−0.2409 (13)	1.92 (1)
O19	0.0925 (9)	0.3490 (5)	0.6984 (10)	1.92 (1)
O20	0.1563 (4)	0.4128 (6)	1.1163 (18)	1.92 (1)
O21	−0.0147 (6)	0.3097 (5)	1.0392 (12)	1.92 (1)
O22	0.0534 (5)	0.1342 (5)	0.8260 (14)	1.92 (1)
O23	0.2350 (6)	0.2817 (5)	0.9225 (11)	1.92 (1)
O24	0.1274 (4)	0.2096 (8)	1.2367 (15)	1.92 (1)
Ow1	0.1040 (6)	0.9740 (9)	0.2663 (9)	3.3 (3)
Ow2	0.5608 (6)	−0.0084 (5)	0.7394 (14)	3.3 (3)
H131	0.4633	0.6655	0.4162	2.32
H132	0.3806	0.5827	0.3264	2.32
H141	0.3599	0.5434	−0.1941	2.32
H142	0.2707	0.5663	−0.1307	2.32
H151	0.2490	0.7339	0.1901	2.32
H152	0.2478	0.8012	0.0019	2.32
H161	0.5276	0.8636	0.2156	2.32
H162	0.4604	0.9084	0.0666	2.32
H171	0.5411	0.6251	0.0349	2.32
H172	0.5500	0.6985	−0.1430	2.32
H181	0.3387	0.7427	−0.3647	2.32
H182	0.4058	0.8405	−0.2709	2.32
H191	0.0576	0.3236	0.5677	2.32
H192	0.0959	0.4217	0.7120	2.32
H201	0.2193	0.4231	1.1642	2.32
H202	0.1328	0.4654	1.1849	2.32
H211	−0.0544	0.2770	1.1307	2.32
H212	−0.0431	0.3594	0.9700	2.32
H221	0.0724	0.0707	0.7991	2.32
H222	0.0102	0.1422	0.7115	2.32
H231	0.2611	0.3158	0.8110	2.32
H232	0.2685	0.2298	0.9610	2.32
H241	0.1823	0.2183	1.3292	2.32
H242	0.0833	0.1608	1.2922	2.32
Hw11	0.1433	0.9720	0.1618	4.0
Hw12	0.1392	0.9761	0.3993	4.0
Hw21	0.6004	−0.0551	0.7197	4.0
Hw22	0.5715	0.0438	0.6432	4.0

All atoms occupy general Wyckoff positions. Atoms of the same type were optimized with identical isotropic displacement parameters. Hydrogen atoms were obtained from geometrical/force-field calculations and not refined (see text). The displacement parameters for the H atoms of the water molecules were coupled to those of the corresponding oxygen atoms according to *B*_iso_(H) = 1.2 × *B*_iso_(O). Occupancies of the zeolitic water positions Ow1 and Ow2 and the associated hydrogen positions have been fixed to 0.9 (see text). All other sites are fully occupied

**Table 3:** Selected individual bond distances (Å) and angles (°) for meta-alunogen

S1-O2	1.4362 (83)	S1-O1	1.4607 (97)	S1-O3	1.481 (11)
S1-O4	1.4840 (84)				
S2-O5	1.478 (10)	S2-O6	1.4705 (95)	S2-O7	1.4625 (84)
S2-O8	1.4616 (87)				
S3-O9	1.4898 (96)	S3-O10	1.4737 (99)	S3-O11	1.4812 (81)
S3-O12	1.504 (10)				
Al1-O16	1.8489 (91)	Al1-O13	1.884 (11)	Al1-O14	1.8884 (98)
Al1-O18	1.888 (10)	Al1-O17	1.906 (12)	Al1-O15	1.9110 (99)
Al2-O20	1.8712 (97)	Al2-O24	1.896 (12)	Al2-O22	1.9130 (84)
Al2-O19	1.9169 (93)	Al2-O23	1.920 (11)	Al2-O21	1.920 (11)
O2-S1-O1	113.35 (60)	O2-S1-O3	109.14 (65)	O2-S1-O4	111.42 (66)
O1-S1-O3	107.40 (813)	O1-S1-O4	106.16 (57)	O3-S1-O4	109.43 (64)
O8-S2-O7	114.54 (66)	O8-S2-O6	107.00 (60)	O8-S2-O5	109.42 (70)
O7-S2-O6	112.88 (66)	O7-S2-O5	107.47 (70)	O6-S2-O5	105.34 (57)
O10-S3-O11	109.99 (59)	O10-S3-O9	106.08 (62)	O10-S3-O12	108.70 (54)
O11-S3-O9	112.99 (58)	O11-S3-O12	104.71 (58)	O9-S3-O12	113.77 (62)

In situ high-temperature X-ray powder diffraction studies have been performed on a Siemens D5005 diffractometer equipped with an Anton Paar HTK1200 furnace. The device has the following specifications: Bragg–Brentano geometry in *θ*–*θ* mode, Cu–*K*α_1/2_-radiation, parallel beam optics, scintillation counter. Moisture*-*dependent powder patterns were obtained using a PANalytical X’Pert PRO diffractometer equipped with a *θ*–*θ* coupled goniometer in transmission geometry, programmable XYZ stage with well plate holder, Cu–*K*α_1/2_-radiation source with a focussing mirror, a 0.5° divergence slit and a 0.02° Soller slit collimator on the incident beam side, a 2 mm anti-scatter slit and a 0.02° Soller slit collimator on the diffracted beam side and a solid-state PIXcel detector. For equilibration of the samples at specific relative humidities, a VGI moisture stage (VGI 2000 M, Middlesex, UK) was used.

### Gravimetric moisture sorption/desorption experiments

Moisture sorption and desorption studies were performed with the automatic multi-sample gravimetric moisture sorption analyzer SPS23-10 µ (ProUmid, Ulm, Germany). Approximately 600 mg of sample was used for the analysis. The measurement cycle was started at 43 % (using a pre-equilibrated alunogen sample, see above), with an initial stepwise desorption (decreasing humidity) to 0 %, followed by a sorption cycle (increasing humidity) up to 80 % and back to 0 % relative humidity. RH changes were set to 5 % for all cycles with the exception of the first step (43 → 40 % RH). The equilibrium condition for each step was set to a mass constancy of ±0.001 % over 60 min and a minimum and maximum time limit of 2 and 48 h, respectively, for each step.

## Results

### Temperature-dependent investigations

**Fig. 2:**
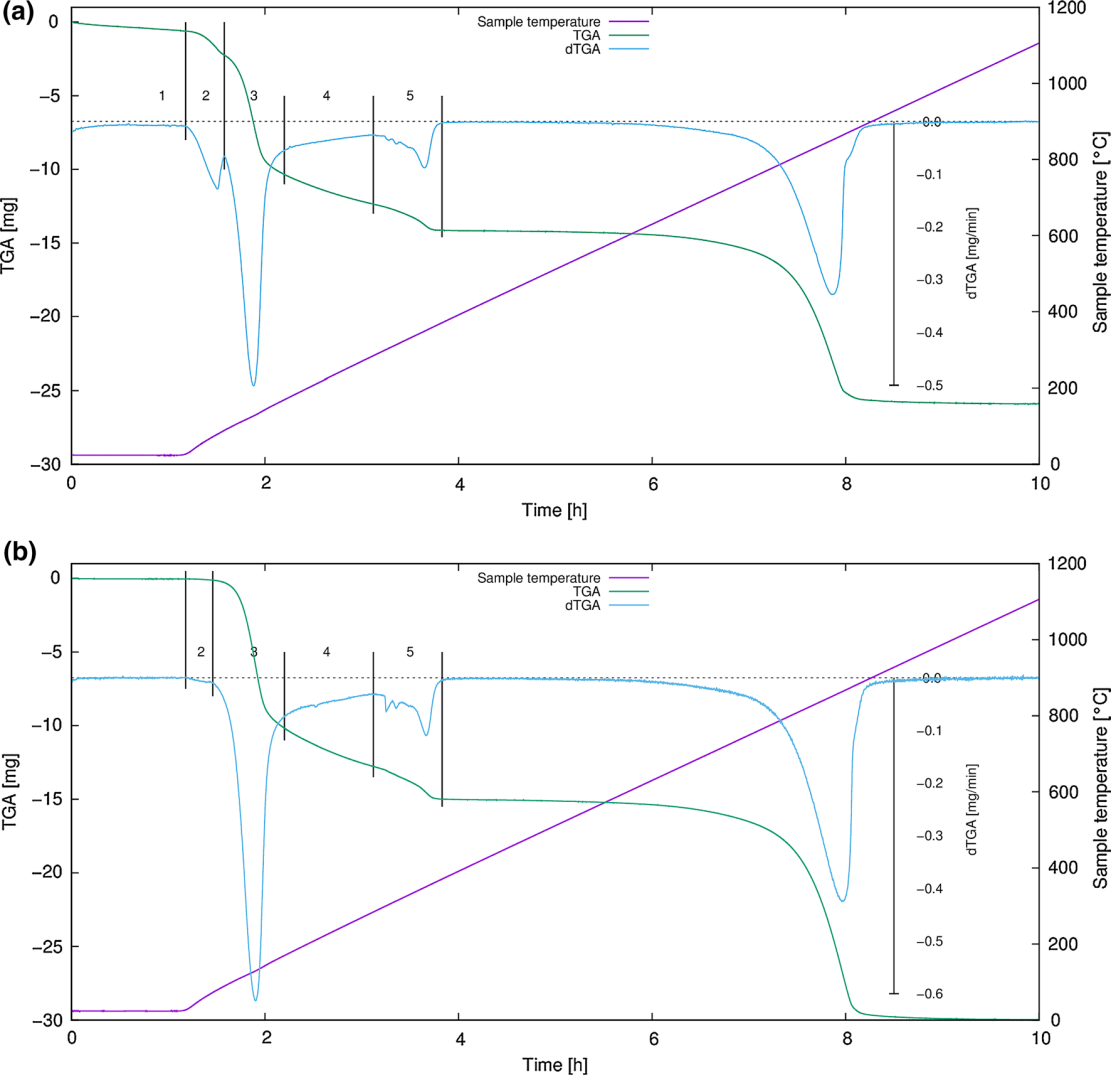
TGA data as a function of time shown together with its first derivative (dTGA) and the sample temperature for alunogen starting materials **a** equilibrated at 75 % RH and **b** stored at 0 % RH over phosphorus pentoxide

The TGA curve and its first derivative (dTGA) look quite similar for the three samples previously stored at 0, 43 and 75 % RH. This is especially true for the samples equilibrated at 43 and 75 % RH, whose curves are almost superimposable. Therefore, only the materials stored at 75 and 0 % are presented/discussed. At any rate the most striking event with a maximum at ca. 850 °C is the decomposition of Al_2_(SO_4_)_3_ into Al_2_O_3_ and SO_3_. The onset of this decomposition is at ca. 800–815 °C (determined from dTGA). In all experiments, the dTGA signal exhibits a small shoulder at the high-temperature side for this effect (see Fig. 2a, b). The dehydration reactions can be subdivided into three to five stages: (1) Samples stored at 75 % RH and 43 % RH show a continuous mass loss at 25 °C, after exposing them to the dry air carrier gas in the thermal analyzer. After 1 h, the loss sums to ca. 0.7 molecules of H_2_O. (2) A small but significant mass loss in the range 25–90 °C is observed for samples equilibrated at 75 % RH and 43 % RH, accounting for approximately two molecules of water. The sample equilibrated at 0 % RH shows a minimal event visible in dTGA only. (3) From ca. 90 °C, a large mass loss step occurs (up to 175 °C). For the 75 % RH and 43 % RH samples, this step corresponds to 9.6 and 9.7 molecules of water, respectively. For the sample stored at 0 % RH over phosphorus pentoxide, this is the *first* significant effect and sums to 9.5 H_2_O. This pronounced dTGA-peak is followed by (4) a continuous, slightly decreasing mass loss rate (up to 290 °C), which accounts for another 2.3 H_2_O molecules. (5) The last dehydration process is observed between 290 and 370 °C: a group of three to four peaks can be identified in the dTGA signal. The mass change corresponds to additional 1.9–2.0 molecules of water. The backward calculation (as described in the experimental section) of the total number of water molecules initially present in the samples resulted in the following values: 75 % RH and 43 % RH: 16.6 molecules per formula unit; 0 % RH: 13.8 molecules per formula unit. These results are in very good agreement with the water contents determined by other methods used in this study. Furthermore, the data indicate that storage under dry conditions already induces the formation of meta-alunogen. Consequently, dehydration steps (1) and (2) occur exclusively for the samples kept at higher relative humidity. We attribute them to a partial loss of zeolitic water from the alunogen structure (effect 1) and the transformation from alunogen to meta-alunogen (effect 2).

**Fig. 3:**
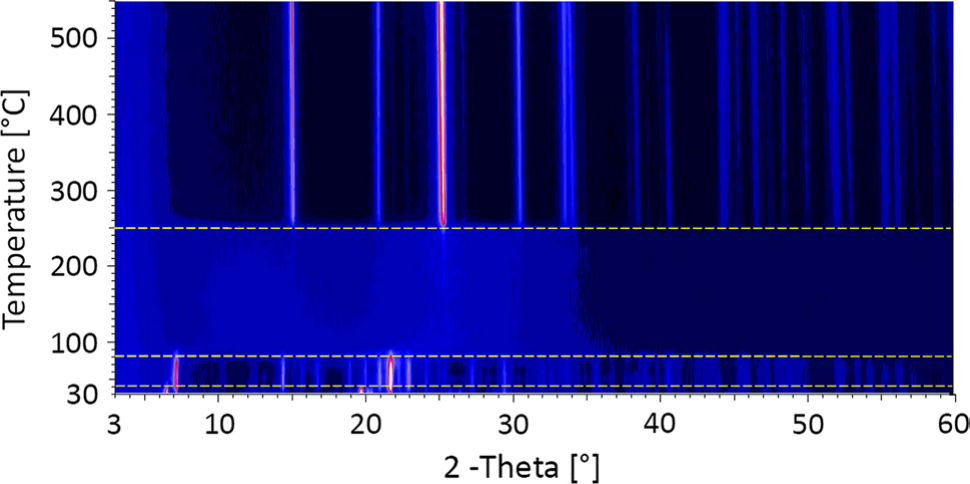
Waterfall plot of the sequence of temperature-dependent X-ray powder diffraction patterns of alunogen the range between 20 and 550 °C collected in situ in steps of Δ*T* = 10 °C

In an in situ X-ray powder diffraction experiment, the dehydration of alunogen equilibrated at 43 % RH has been studied in the range between 20° and 550 °C. Diffraction patterns have been collected every 10 °C using the following parameters: scan step size: 0.02° 2*θ*; counting time per step: 12 s; ramp rate between set points: 0.2 °C/sec; 900 s dwell time at the set points before starting the measurement; total data collection time per scan: 9 h 45 min. A waterfall plot summarizing the results is given in Fig. 3. According to these measurements, the thermal stability of the alunogen structure is rather limited. At a temperature of about 40 °C, alunogen already started to decompose. At 50 °C no peaks of the original alunogen sample could be detected any more, i.e., the recorded X-ray powder pattern belonged to a new crystalline phase, which is stable up to 80 °C. No significant differences between the full-width-at-half-maximum values of both phases could be observed, i.e., the crystallite sizes do not seem to be much affected by the transformation. At 90 °C a further reaction can be monitored resulting in the complete amorphization of the material. The sequence of “amorphous humps” in the patterns persists up to 250 °C, where a re-crystallization process is indicated by a sudden appearance of a larger number of sharp Bragg peaks. Phase analysis confirmed this compound to be anhydrous Al_2_(SO_4_)_3_. However, a more detailed analysis of the data reveals that the onset of the strongest reflection of aluminum sulfate can be already seen at 170 °C. This points to a simultaneous presence of a hydrous amorphous material, and a much smaller amount of Al_2_(SO_4_)_3_ of low crystallinity. Al_2_(SO_4_)_3_ remains stable up to 550 °C, corresponding to the maximal temperature of this in situ diffraction experiment. Shifts of the lines in the range between 250 and 550 °C are due to thermal expansion.

**Fig. 4:**
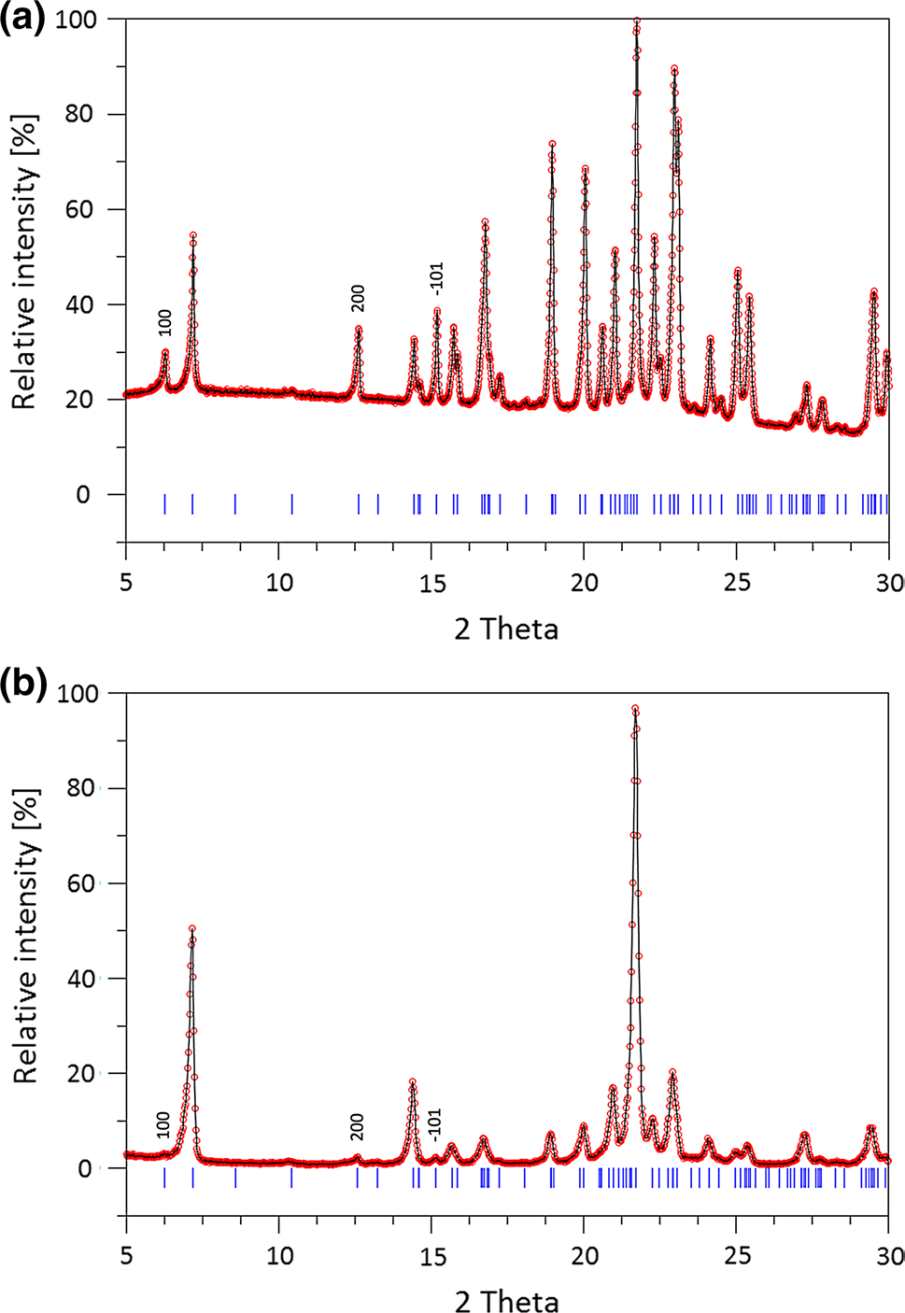
Low angle regions of the powder diffraction patterns of meta-alunogen samples obtained from alunogen by **a** storing over phosphorus pentoxide at 0 % RH (Debye–Scherrer transmission geometry, Cu–*K*α_1_) and **b** in situ dehydration at 60 °C (Bragg–Brentano reflection geometry, Cu–*K*α_1/2_). *Vertical tick marks* indicate the allowed peak positions for the triclinic unit cell of meta-alunogen derived from ab-initio indexing. For sake of clarity only the lines for Cu–*K*α_1_ radiation are plotted. Differences in relative intensities are due to preferred orientation (see text)

The comparison between the powder patterns of the samples (1) stored (ex situ) at 0 % RH and (2) acquired (in situ) at 60 °C and is given in Fig. 4a, b. Notably, at a first glance, the diffractograms look quite different. However, both patterns can be satisfactorily explained using the same above-mentioned triclinic unit cell of meta-alunogen. Differences in intensities can be attributed to a pronounced preferred orientation of the “in situ sample” that has been collected in Bragg–Brentano geometry. Although the exact morphology of the crystals of meta-alunogen has not been studied, the structural similarities with alunogen (which will be discussed later) suggest that the platy shape and the perfect cleavage typical for alunogen is retained. The platy morphology reflects the presence of layer-like building elements running parallel to (010) of the triclinic cell of meta-alunogen. Therefore, in reflection mode, the (h0l)-peaks show a pronounced reduction in intensity.

### Humidity-dependent investigations

Based on the moisture sorption/desorption behavior hydrates can be divided into two main classes, the *stoichiometric* and *non*-*stoichiometric* hydrates (Gál 1968). Stoichiometric hydrates show step-shaped sorption/desorption isotherms characterized by a fixed water content over a defined relative humidity range and generally convert upon dehydration to a distinct phase. In contrast, non-stoichiometric hydrates have a continuously variable composition within a certain range that is not associated with a significant change in the crystal structure, except some anisotropic expansion of the network to accommodate the water.

**Fig. 5:**
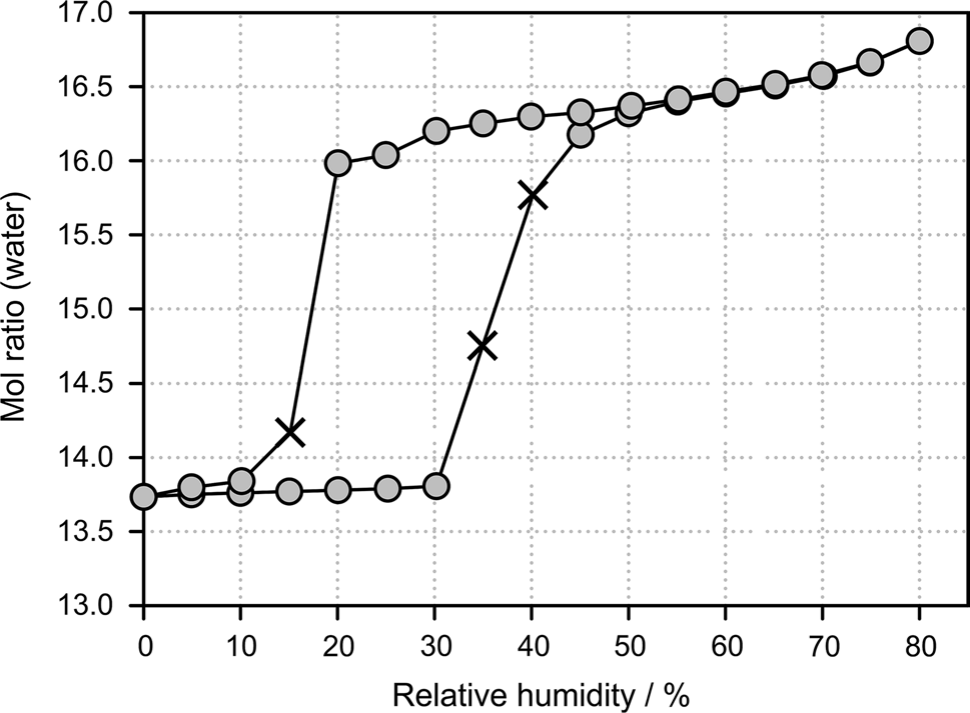
Gravimetric moisture sorption and desorption curves of alunogen at 25 °C. The *gray circles* represent data points reaching the equilibrium (constant mass) within the pre-set maximal equilibration time, whereas crosses mark data points where the sample did not reach the equilibrium moisture content within the allowed time limit of 48 h

The water sorption and desorption characteristic of alunogen was investigated between 0 and 80 % RH at 25 °C (Fig. 5). The lowest water content measured for alunogen at 0 % RH corresponded to 13.7 mol of water per mol Al_2_(SO_4_)_3_. Upon increasing the RH from 0 to 30 %, almost no water uptake was seen, i.e., less than 0.1 mol equivalent of water was sorbed and no phase transformation was observed. However, on further increasing the RH (>35 %), a phase transition associated with a mass increase of max. 3.1 mol water per mol Al_2_(SO_4_)_3_ was observed. The product phase, confirmed to be a distinct hydrate phase by XRPD, showed at 80 % RH a maximum water to compound ratio of 16.8:1. Upon further increasing the RH, deliquescence of the material was observed. The desorption isotherm shows the typical course of a non-stoichiometric hydrate in the RH range between 80 and 20 %, which is indicated by the mass gradually changing depending on the humidity. The higher hydrated phase (alunogen) can accommodate mol-equivalents of water ranging from 16.8 (80 %) to 16.0 % (20 %). At RH values below 20 % a distinct step, corresponding to a mass loss of about 2 mol of water per mol Al_2_(SO_4_)_3_ was measured, resulting in the lower hydrated 13.7-hydrate corresponding to meta-alunogen. Hysteresis was observed in the meta-alunogen/alunogen isotherms, despite the lengthy equilibration times (up to 48 h) at each RH. The observed hysteresis and distinct steps in the sorption/desorption isotherms are characteristic for a stoichiometric behavior. Thus, only RH-dependent methods allowed to qualify the stoichiometric and non-stoichiometric hydration/dehydration behavior of alunogen.

**Fig. 6:**
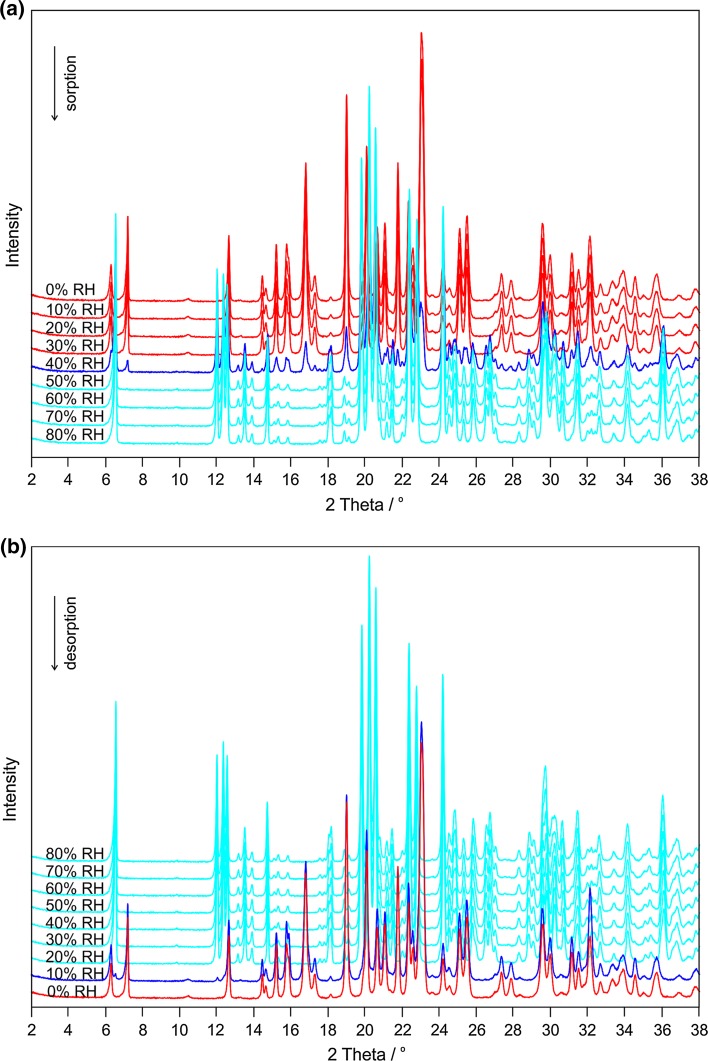
Moisture-dependent XRPD measurements of alunogen recorded at 25 °C in the RH range **a** between 0 and 80 % (sorption) and **b** between 80 and 0 % (desorption). Hysteresis between the transformations from meta-alunogen to alunogen (at about 40 % RH, sorption) and from alunogen to meta-alunogen (at about 20 % RH, desorption) is clearly visible

The humidity-controlled XRPD investigations performed at 25 °C correlate well with the gravimetric moisture sorption/desorption studies. In agreement with the results presented in Fig. 5, distinct changes occur in the XRPD patterns of the sample at RH values >30 % on increasing the RH (Fig. 6a, sorption) and at RH values <20 % on decreasing the RH (Fig. 6b, desorption). A major change in the crystal structures is indicated by the appearance and disappearance of characteristic reflections, which correspond to the reaction (dehydration) from alunogen to meta-alunogen and vice versa. In contrast, at RH values >40 % RH (sorption) and ≥20 % (desorption), there is no significant change in the crystal structure apart from anisotropic effects to accommodate a variable amount of water.

### Crystal structure of meta-alunogen

The asymmetric unit of meta-alunogen contains three symmetry independent SO_4_-tetrahedra and two Al(H_2_O)_6_-octahedra. The polyhedra are isolated, however, linkage between them is provided by Coulomb interactions and hydrogen bonding. In addition to the water molecules which directly belong to the coordination environment of the aluminum cations, there are two additional zeolitic water sites (Ow1 and Ow2). If both positions are fully occupied, meta-alunogen corresponds to a 14-hydrate. However, similar to alunogen, the meta-alunogen structure represents a non-stoichiometric hydrate where the water content can vary as a function of the occupancies of the interstitial H_2_O-sites. Due to strong correlations between their site occupancies and isotropic displacement factors, we refrained from a simultaneous refinement but tried to optimize them by a sequence of strictly alternating refinements, which indeed pointed to a deficiency of the water populations on both sites. Therefore, we finally decided to fix the occupancies of the zeolitic water positions to 90 % resulting in a total water content of 13.8 moieties p.f.u. and corresponding to the value obtained in the TGA experiments. The quality of our in-house diffraction data did not allow a location of the hydrogen positions of the water molecules from standard methods such as difference Fourier synthesis. To gain at least some insight into the hydrogen bonding scheme of meta-alunogen approximate positions of the H atoms were obtained by combined geometric and force-field calculations on the basis of hydrogen bonding interactions using the program CalcOH (Nardelli 1999) implemented as a GUI version within the WinGX software suite (Farrugia 2012). For the calculation of the hydrogen atoms of the water moieties, a d_O–H_ target value of 0.90 Å was used. The table summarizing the hydrogen bonds is given as supplementary data.

The values for the S–O distances are in the normal range for sulfates (Hawthorne et al. 2000). The range of the individual S–O bond distances for the three tetrahedra is between 1.44 and 1.50 Å (average: 1.474 Å). The O–S–O angles vary between 104.7 and 114.5°. Al–O distances are in the range between 1.849 and 1.920 Å. Bond valence sums in valence units (v.u.) for the cations were calculated using the bond valence parameters of Brown and Altermatt (1985). The resulting values are 6.16 (S(1)), 6.09 (S(2)), 5.80 (S(3)), 3.02 (Al(1)) and 2.77 (Al(2)) v.u. and thus are reasonably close to the ideal values.

**Fig. 7:**
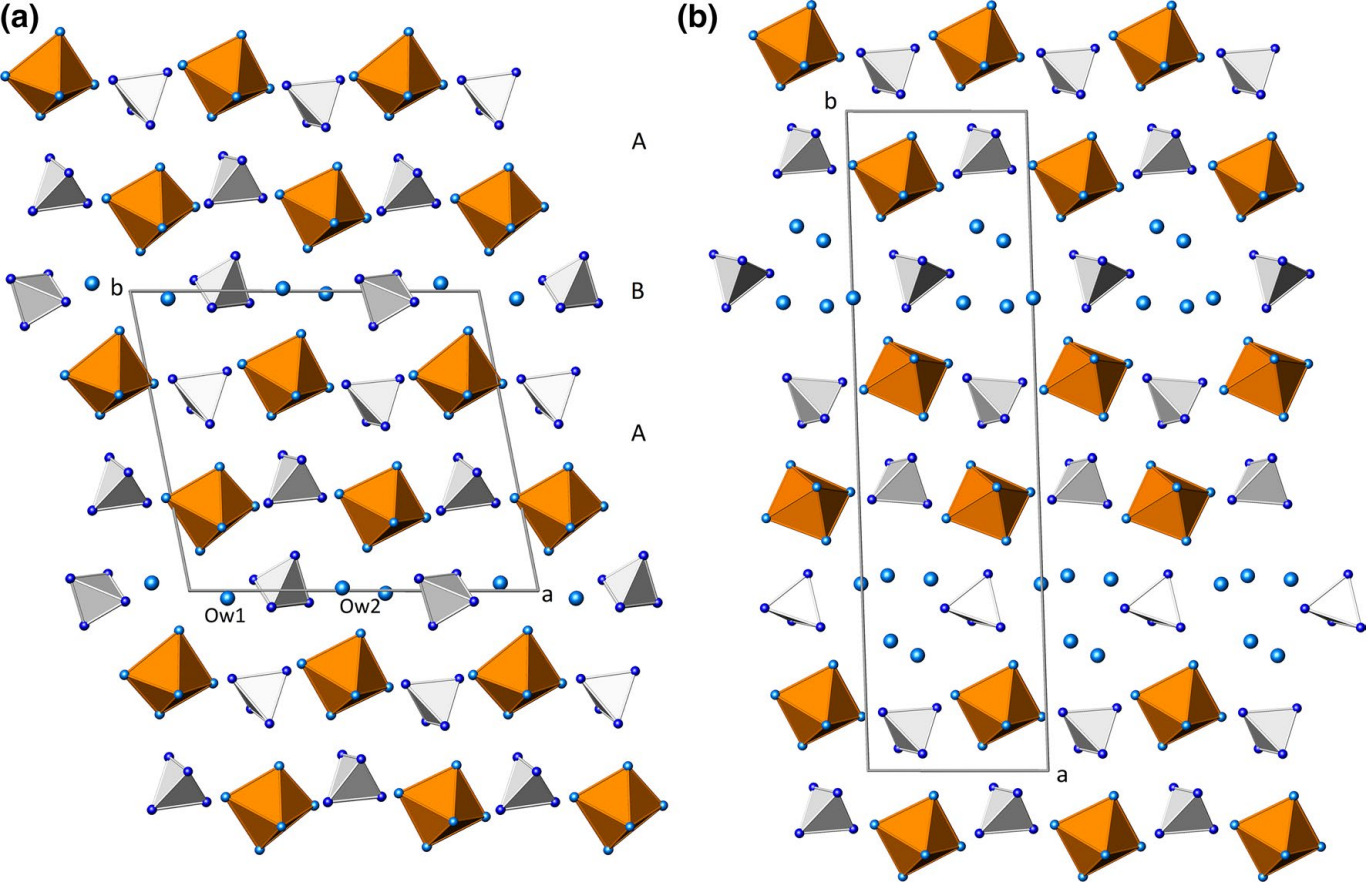
Crystal structures of **a** meta-alonogen and **b** alunogen in projections parallel to the slabs containing the polyhedral units. *Small dark* and *light blue spheres* represent oxygen atoms of the sulfate groups and of water molecules belonging to the coordination environment of the aluminum atoms, respectively. *Larger light blue spheres*: zeolitic water positions (color figure online)

A projection of the whole structure of meta-alunogen parallel to [001] is given in Fig. 7a. As is obvious from this figure, the structure can be divided into two layers running parallel to (010). Layer type B is centered at *y* ≈ 0 and contains the tetrahedra around S3 and the zeolitic water molecules. The second layer (type A) located at *y* ≈ ½ contains the S1O_4_- and S2O_4_-tetrahedra as well as the Al(H_2_O)_6_-octahedra. Within slab-type A, chains of (unconnected) alternating octahedra and tetrahedra can be identified which are running parallel to [100]. The layers are stacked in a …ABAB… sequence along the *b*-axis. Notably, the same layer types can be also found in alunogen (Menchetti and Sabelli 1974; Fang and Robinson 1976). However, in alunogen, more complex …ABA′B′… sequences are realized (see comparison between Fig. 7a, b). Actually, in alunogen, adjacent A or B slabs are related by inversion centers, whereas in meta-alunogen the corresponding structural units are already translationally equivalent leading to a reduction of the stacking period by roughly a factor of two. Further shrinkage along this direction is due to the release of approximately three water molecules p.f.u. resulting in a final change of the *b* lattice parameter from 26.975 Å in alunogen to 12.490 Å in meta-alunogen. In contrast, the value for the *a* unit-cell parameter in meta-alunogen is about two times bigger than in alunogen (14.353 Å and 7.425 Å, respectively). As is obvious from Fig. 7a and b, an increased amount of shear between neighboring AB-packages can be observed in meta-alunogen which is reflected in an increase of the *γ*-angle from about 91.9 (alunogen) to 100.8° (meta-alunogen).

## Discussion

Although both alunogen and meta-alunogen exhibit a certain degree of flexibility in their exact water contents, there is a sharp transition between the two phases that can be unambiguously followed using the in situ diffraction studies. The transformation is also evident from the thermo-analytical investigations. Variability between the particular transition temperatures is probably due to pronounced differences in the heating rates employed in both techniques. The TGA studies indicate numerous dehydration steps; however, these distinct events cannot be observed in the diffraction experiments. The reason therefore can be seen in the fact that the release of strongly bound water molecules from the meta‐alunogen structure is combined with a breakdown of long‐range order. The complex temperature- and moisture-dependent dehydration behavior of alunogen to meta-alunogen is another prime example, highlighting that the combination of different analytical techniques is a prerequisite for a holistic understanding of dehydration reactions.

Using the Magnus equation (Buck 1981) for an estimation of the saturation water vapor pressures (*p*_S_) for the temperature range between 5 and 50 °C (which may be considered to be representative for the range of surface temperatures of the alunogen localities on Earth) results in values for p_S_ between 8.7 and 123.5 mbar. They are much lower, for example, than the value of 1.0123 bar (= 1 atm) for total water vapor pressure that was used by Gancy (1981) during his experiments to synthesize *crystalline* Al_2_(SO_4_)_3_·9H_2_O and Al_2_(SO_4_)_3_·5H_2_O from dehydration of a synthetic alunogen sample. Furthermore, single crystals of lower hydrates such as Al_2_(SO_4_)_3_·5H_2_O, Al_2_(SO_4_)_3_·8H_2_O and Al_2_(SO_4_)_3_·10.5H_2_O (Fischer et al. 1996a, b, c) were obtained by evaporation of solutions of alunogen in sulfuric acid at temperatures between 110 and 130 °C followed by a hydrothermal treatment. Within this context, it is interesting to note that Witzke et al. (2015) only recently reported the existence of an unnamed mineral with composition Al_2_(SO_4_)_3_·5H_2_O from the Anna I burning coal mine dump (Aachen region, Germany). This new mineral is associated with millosevichite and godovikovite (NH_4_Al(SO_4_)_2_) in pumice-like aggregates formed in hotter vents (140–200 °C). According to an unpublished powder diffraction study, it represents the natural analogue to the above-mentioned synthetic pentahydrate (T. Witzke, personal communication, June 6, 2016). In summary, these observations indicate that under temperature and moisture conditions at the Earth’s surface, meta-alunogen is probably the only *crystalline* dehydration product that can be obtained from alunogen directly.

Notably, alunogen is at the edge of its stability even at ambient conditions. Slightly elevated temperatures and/or relative humidities below 20 % can induce the release of some of the loosely bound water molecules triggering a phase transformation. Apart from alunogen, there are several other hydrous minerals known showing a similar behavior. Examples include the group of magnesium sulfates (Chipera and Vaniman 2007; Mills et al. 2010) or kanemite (NaSi_2_O_4_)(OH)·3H_2_O, Schmidmair et al. 2015). Especially the influence of humidity on the stability of minerals has not been studied in great detail. However, a thorough understanding of this parameter is a prerequisite for finding the best way to store mineral specimens in a museum collection so that they can be preserved.

**Fig. 8:**
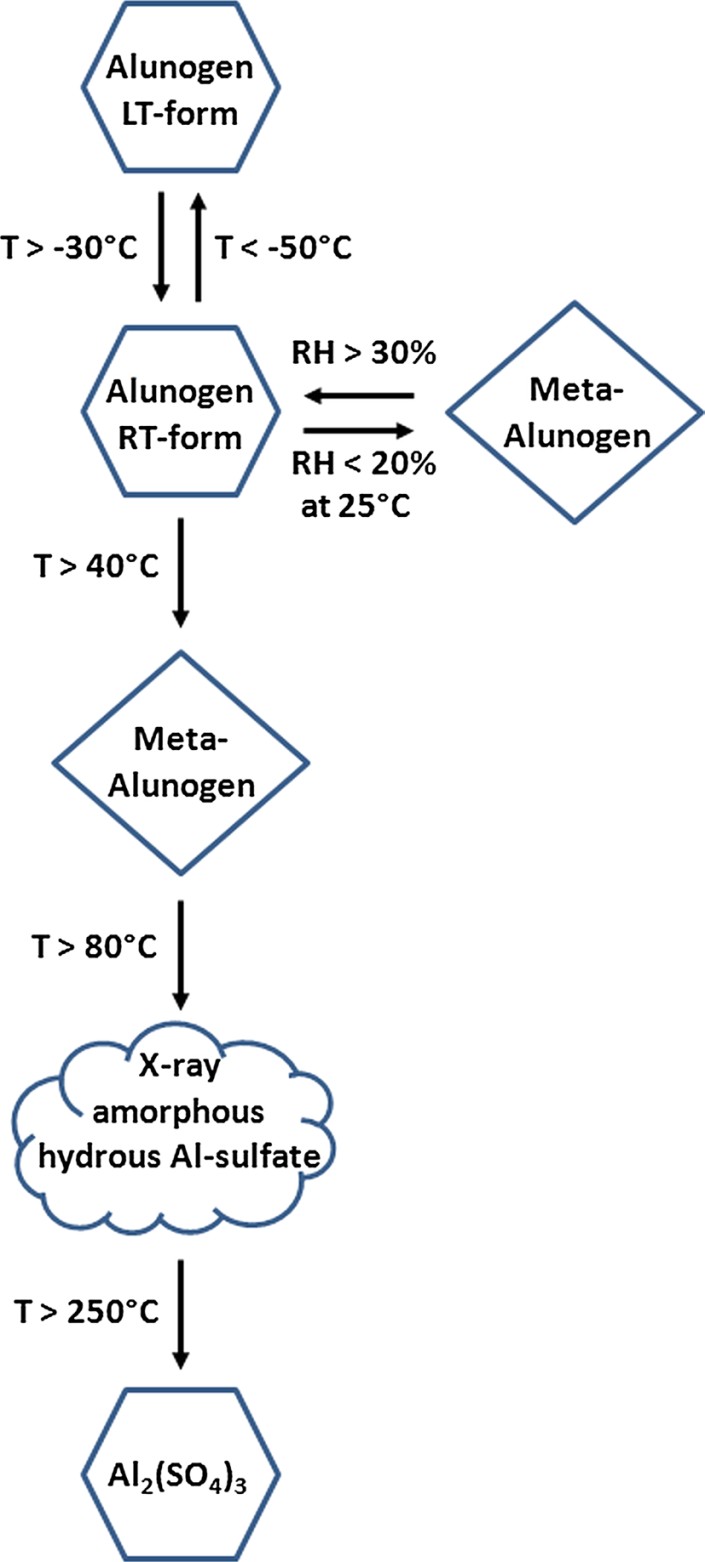
Summary of the temperature- and moisture-dependent behavior of alunogen based on the diffraction studies of the present paper and the results published by Kahlenberg et al. (2015)

Furthermore, the reported structural model for meta-alunogen can be used for quantification of this mineral in polycrystalline multi-phase mixtures using the Rietveld method. Since meta-alunogen has been postulated to exist on the surface of Mars (Golden et al. 2005; Wang and Zhou 2014), our new improved powder diffraction data may also help in the identification of this mineral by X-ray diffraction of Martian soils using the Curiosity Rover’s ChemMin instrument (Bish et al. 2014). We would like to mention that the present study completes our knowledge concerning the response of alunogen to temperatures different from ambient conditions. Only recently, our group was able to show that alunogen undergoes a first order transition somewhere between −30 and −50 °C from the triclinic room-temperature form to a monoclinic LT-polymorph (Kahlenberg et al. 2015). A scheme summarizing the temperature- and moisture-dependent behavior of alunogen is given in Fig. 8. Concluding, one can say that the results of this investigation clearly correct a statement in the paper of Bayliss (1964) claiming that meta-alunogen is not a distinct mineral but merely a partly dehydrated form of alunogen.

## Electronic supplementary material

Below is the link to the electronic supplementary material.

Supplementary material 1 (PDF 45 kb)
