# Supplementary material for: Temperature- and moisture-dependent studies on alunogen and the crystal structure of meta-alunogen determined from laboratory powder diffraction data
Source: Phys Chem Miner. 2016 Sep 19;44(2):95–107. doi: 10.1007/s00269-016-0840-7 (PMC5306069; doi:10.1007/s00269-016-0840-7)
Supplement: Supplementary file 1 — Supplementary material 1 (PDF 45 kb) [file 269_2016_840_MOESM1_ESM.pdf]

Hydrogen bonds within the structure of meta-alunogen resulting from the predicted hydrogen positions: distances in (Å) and angles in (°).

| D-H...A | d(D-H) | d(D...A) | d(H...A) | <(DHA) |
|---|---|---|---|---|
| O13 -H131 ...O3 | 0.900 | 2.506 | 1.625 | 165.38 |
| O13 -H132 ...O1 | 0.900 | 2.495 | 1.619 | 163.36 |
| O14 -H141 ...O4 | 0.900 | 2.462 | 1.611 | 156.47 |
| O14 -H142 ...O6 | 0.900 | 2.834 | 2.030 | 147.97 |
| O15 -H151 ...O7 | 0.900 | 3.229 | 2.474 | 141.77 |
| O15 -H151 ...O8 | 0.900 | 2.959 | 2.124 | 153.96 |
| O15 -H152 ...O10 | 0.900 | 2.693 | 1.886 | 148.10 |
| O16 -H161 ...O11 | 0.900 | 3.080 | 2.335 | 140.08 |
| O16 -H161 ...O12 | 0.900 | 3.316 | 2.649 | 131.68 |
| O16 -H162 ...O18 | 0.900 | 2.687 | 2.494 | 92.46 |
| O16 -H162 ...Ow2 | 0.900 | 2.090 | 1.771 | 97.55 |
| O17 -H171 ...O4 | 0.900 | 2.786 | 1.945 | 154.74 |
| O17 -H172 ...O3 | 0.900 | 2.614 | 1.799 | 149.38 |
| O18 -H181 ...O8 | 0.900 | 2.309 | 1.562 | 137.69 |
| O18 -H182 ...O12 | 0.900 | 2.553 | 1.965 | 121.50 |
| O18 -H182 ...Ow2 | 0.900 | 3.440 | 2.626 | 150.95 |
| O19 -H191 ...O5 | 0.900 | 2.727 | 1.950 | 143.64 |
| O19 -H192 ...O6 | 0.900 | 2.611 | 1.806 | 147.52 |
| O20 -H201 ...O1 | 0.900 | 2.830 | 1.949 | 165.79 |
| O20 -H201 ...O23 | 0.900 | 2.485 | 2.307 | 90.61 |
| O20 -H202 ...O7 | 0.900 | 2.798 | 1.925 | 162.92 |
| O21 -H211 ...O5 | 0.900 | 2.492 | 1.755 | 137.25 |
| O21 -H212 ...O7 | 0.900 | 2.630 | 1.827 | 147.44 |
| O22 -H221 ...O9 | 0.900 | 2.838 | 2.173 | 130.23 |
| O22 -H222 ...O19 | 0.900 | 2.801 | 2.640 | 90.82 |

| | | | | | |
|---|---|---|---|---|---|
| O22 | -H222 ...Ow1 | 0.900 | 2.395 | 1.997 | 105.12 |
| O23 | -H231 ...O2 | 0.900 | 2.583 | 1.814 | 141.87 |
| O23 | -H231 ...O4 | 0.900 | 3.145 | 2.367 | 144.74 |
| O23 | -H232 ...O11 | 0.900 | 2.604 | 1.765 | 153.90 |
| O24 | -H241 ...O2 | 0.900 | 2.617 | 1.777 | 154.19 |
| O24 | -H242 ...Ow1 | 0.900 | 2.915 | 2.406 | 115.96 |
| Ow1 | -Hw11 ...O10 | 0.900 | 2.677 | 1.788 | 168.94 |
| Ow1 | -Hw12 ...O9 | 0.900 | 2.463 | 1.628 | 152.81 |
| Ow2 | -Hw21 ...O11 | 0.900 | 3.104 | 2.636 | 113.23 |
| Ow2 | -Hw22 ...O12 | 0.900 | 3.168 | 2.544 | 126.93 |
| Ow2 | -Hw22 ...O16 | 0.900 | 2.090 | 1.883 | 90.18 |
